# EQ-5D Brazilian population norms

**DOI:** 10.1186/s12955-021-01671-6

**Published:** 2021-06-10

**Authors:** Marisa Santos, Andrea L. Monteiro, Bráulio Santos

**Affiliations:** 1 Nucleo de Avaliacao de Tecnologias Em Saude, Instituto Nacional de Cardiologia, Rio de Janeiro, Brazil; 2 Department of Pharmacy Systems, Outcomes and Policy, College of Pharmacy, University of Illinois at Chicago, Chicago, IL USA

**Keywords:** Health-related quality of life, EQ-5D-3L, Population norms, Brazil

## Abstract

**Background:**

The EQ-5D-3L is a widely used generic health-related quality of life measure commonly applied to describe health outcomes and to measure disease burden. The aim of this study was to generate Brazilian population norms, stratified by age and gender, based on Brazilian preference weights for EQ-5D-3L.

**Methods:**

A multicenter cross-sectional study was conducted in three Brazilian urban areas. The final sample consisted of 5774 respondents, aged from 18 to 64 years. Amongst other information, respondents were asked to self-report their health status using the EQ-5D-3L descriptive system and visual analog scale (EQ-VAS). Data on socio-demographic characteristics was obtained through specific questionnaires. The Brazilian TTO scoring algorithm was used to derive the utility values. Multivariate logistic regression models were fitted to analyze the influence of age, sex, education status and sample site on the presence of any problem for each dimension of EQ5D.

**Results:**

Mean values were computed for both weighted index scores and self-rated health status (EQ-VAS), and stratified by gender and age groups. Health status declines with age, ranging between 0.87 for the youngest group 18–29 year-olds and 0.76 for 60–64-year-old. Men reported higher scores (0.85) than the woman (0.79). Lower education levels were associated with lower EQ-5D index score in most age groups.

**Conclusion:**

This study provides EQ-5D reference values for the Brazilian population. These values can be used by local decision-makers and researchers in economic evaluations and population health studies.

## Background

High-level health care policy planning must rely on relevant information about the health state of patient groups as well as the preferences of the general population. Over the course of the last couple of decades, quality of life (QoL) has emerged amongst the measurable outcomes of health programs and interventions as an appropriate outcome to account for actual improvements on patient’s overall health status [1]. Generic QoL measures are generally composed of items that summarize different aspects of individual health status, such as symptom relief, mental and physical function [2–4]. Given its focus on common core characteristics of health, generic measures are suited to assess the impact of a treatment on a specific condition and to enable comparisons across different conditions. For instance, in order to compare interventions and health programs within the same condition (with diverse effects on both symptoms relief and physical function, e.g. ‘pain-free days’ and ‘ability to carry on daily activities’) and across different conditions (with diverse effects on non-comparable outcomes, as for instance ‘disease-free survival’ and ‘incidence of femur fractures’) it is useful to resort to an outcome measure that captures not only the effects on clinical events but also the effects on their overall quality of life [5, 6].

Among generic measures of QoL, one can find the generic preference-based measures. These instruments describe health states that can be weighted according to preference values derived by scoring functions [7]. The flagship application of generic preference-based measures is to measure and value health outcomes so as to assign values to health changes and inform cost-effectiveness analysis, specifically those that rely on the cost-utility framework [8]. On cost-utility analysis, the additional benefits produced by a new intervention are expressed in terms of quality-adjusted life-years (QALYs), which results in an incremental cost-effectiveness estimate presented as cost per QALY gained. The QALY comprises, in a single index, the measure of a person’s length of life weighted by a valuation of their health-related quality of life [7, 9, 10]. The utility values used in QALY calculations are usually elicited on valuation studies conducted with patients or general population (value sets), wherein the health states derived from preference-based measures are valued [2, 5]. Other strategies to obtain these weights include different approaches and populations (e.g., healthcare professionals' opinions; non-preference based weights; mapping) may be used, but are considered suboptimal [6].

When computing QALY estimates, the time spent in a given health state (estimated survival) is multiplied by the utility weight correspondent to that health state. The utility weight represents how much years of life a person is willing to sacrifice to improve their QoL from a specific health state.

Preference-based measures can be used as “off-the-shelf” outcome measures to assess QoL within a clinical trial or observational study, providing utility values as an outcome. Other applications for preference-based measures include the measurement of population health and disease burden.

This paper will focus on the population norms derived from self-reported QoL data collected during the Brazilian valuation study of the EQ-5D-3L. The EQ-5D is a widely used generic preference-based measure composed by a descriptive system and a visual analog scale (VAS) [11]. Thus far, there are three versions of the EQ-5D: the three and five level versions of the adult-oriented descriptive systems, and the EQ-5D Youth (EQ-5D-Y), developed to serve as an outcome measure for children and adolescents. The EQ-5D descriptive systems comprise five domains: mobility, self-care, usual activities, pain/discomfort and anxiety/depression. In the three level version, each of the five dimensions can be described with three levels of problems (no problems, some/moderate problems, and extreme problems). This descriptive system defines 243 different health states (3^5^), wherein each one of them can be described in a 5-digit profile that combines information about the level of problems in each dimension (e.g., no problems in all dimensions ‘11111’; extreme problems in all dimensions ‘33333’) [12]. The validity and reliability of the EQ-5D-3L have been well documented elsewhere [2, 13].

To date, several countries, among them the U.K. [14], USA [15, 16], Poland [17] Portugal [18], Denmark [19], Singapore [20, 21], and China [22], have made their population norms for the EQ-5D available. Furthermore, Brazilian data on the use of the EQ-5D-3L as a health measure for the adult population has been recently published [23]. Once data of this nature is made available, it can be used to inform the interpretation of quality of life estimates, measured by the EQ-5D-3L. The existence of normative data enables comparisons between data collected from specific groups (e.g., patients, occupational groups, ethnic groups) with the general population, making it possible, for instance, to explore weather a specific condition is associated with quality of life impairments. This study aims to generate Brazilian population norms for EQ-5D-3L, stratified by age and gender.

## Methods

### Survey design and data collection

The data used in this analysis was collected during the national valuation study of the EQ-5D-3L in Brazil. A total of 5774 respondents from the general public were interviewed in mid-2012. The sample was drawn from three Brazilian urban centers (Rio de Janeiro, Porto Alegre, and Recife). The sampling frame was established based on data from the Brazilian 2010 census. The states from which data were collected represent 4/3 of the seven most populous Brazilian states, accounting for approximately 30% of the total population.

A geographically based probabilistic sample of the general population from urban areas, with ages ranging from 18 to 64 and stratified by age and gender was recruited and interviewed. Potential participants were targeted resorting to area-based measures derived from Brazilian census tract records, and eligible subjects were identified following a door-to-door household recruitment strategy. Due to the Brazilian territorial dimension, and to the budgetary constraints typical to a research project exclusively funded with public resources, the research team chose to restrict the data collection to urban settings. The implications of this choice on the representativeness of the results will be further debated in the discussion section.

Given that the valuation of the EQ-5D-3L health states was the primary purpose of the main study that generated this dataset, the sample size estimation was based on the desired number of observations for each pair of health states (at least 140 for each). Further details on this survey and its design have been previously reported by Santos et al. [24]. The variables were selected based on the conceptual model based on the literature.

EQ-5D -3L was selected because it is the only Euroqol instrument with Brazilian Valuation data. EQ-5D Index Values (utility values) are obtained thought a scoring function from a set of population-based preference weights from the Brazilian valuation study [24].

### Socio-economic component

In addition to the valuation and self-rating tasks, the respondents also completed a questionnaire about their socio-economic characteristics. Each respondent was asked about gender, age, religious beliefs, marital status, education level, and ownership of goods. The socio-economic classification used in this study was based on the classification proposed by ABEP—Brazilian Association of Survey Companies in 2013 [25]. This approach combines data on ownership of goods and education level to create an eight-level classification system, A corresponds to the wealthiest class defined (with estimated average yearly earnings of US$4,988), and the classes D/E are the most impoverished strata of this classification system (with estimated yearly earnings of US$376).

### Statistical analysis

The statistical analysis was conducted using STATA 14 (StataCorp L.P., Stata Statistical Software; TX, USA). The distribution of the utility estimates, median utility, and inter-quartile range (IQR) were computed and stratified by sample characteristics. Categorical variables were described as count (proportion) and continuous variables as mean (standard deviation) or median (interquartile range), depending on the variable distribution. We used multivariate logistic regression models to analyze the influence of age, sex, education status and sample site on the presence of any problem for each dimension of EQ5D. Results were displayed as odds ratio and 95% confidence intervals. This analysis was conducted using unweighted data.

## Results

This analysis was performed using data from 5774 respondents. Our respondents were young (mean age was 38 years), married (about 51%), with children (about 68%). A distinctive aspect of this sample comparing to international data is that almost 34% of respondents had primary education or less. The respondents, for the most part (97%), believed in God. Detailed sample characteristics are presented in Table 1.

**Table 1:** Sample characteristics

Factor	Level	Value
N		5774
City	Rio de Janeiro	3921 (67.91%)
	Recife	959 (16.61%)
	Porto Alegre	894 (15.48%)
Sex	Female	3104 (53.76%)
	Male	2670 (46.24%)
Age, median (IQR)		38 (27, 49)
Marital status	Married	2925 (50.66%)
	Widowed	182 (3.15%)
	Divorced	488 (8.45%)
	Single	2176 (37.69%)
	Didn't know/didn't answer	3 (0.05%)
Children	No	1826 (31.62%)
	Yes	3948 (68.38%)
ISCED* level of education (2011)	0–2	1934 (33.49%)
	3–4	2166 (37.51%)
	5–6	1302 (22.55%)
	7–8	372 (6.44%)
Socio-economic status**	A	383 (6.63%)
	B	2370 (41.05%)
	C	2767 (47.92%)
	D/E	239 (4.14%)
	Didn't know/didn't answer	15 (0.26%)
Chronic disease	No	2183 (37.81%)
	Yes	3591 (62.19%)
Health insurance	No	3215 (55.68%)
	Yes	2554 (44.23%)
	Didn't know/didn't answer	5 (0.09%)
Believes in God	No	150 (2.60%)
	Yes	5601 (97.00%)
	Didn't know/didn't answer	23 (0.40%)
Self-rated health	Very good	1093 (18.93%)
	Good	2863 (49.58%)
	Fair	1639 (28.39%)
	Bad	139 (2.41%)
	Very bad	38 (0.66%)
	Didn't know/didn't answer	2 (0.03%)
VAS, median (IQR)		85 (70, 90)

*0–2 from no formal to lower secondary education. 3–4 upper to post-secondary, 5–6 tertiary education, 7–8 post-graduation

**Socio-economic status A/B the wealthiest class

**Fig. 1:**
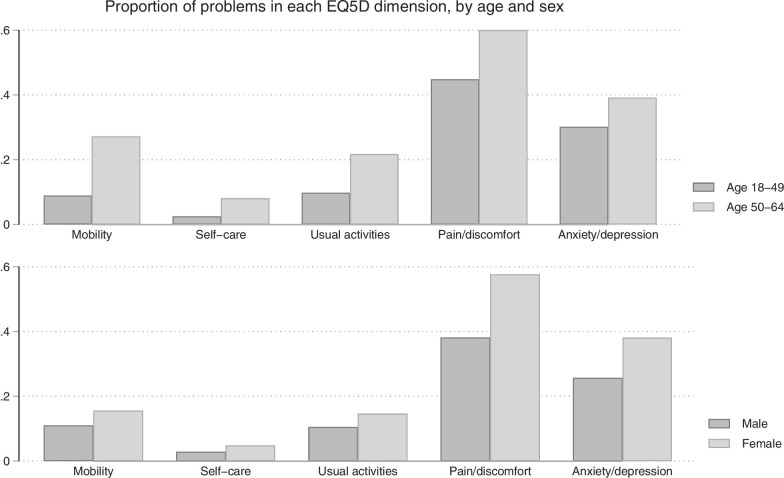
Percentage of reported problems for each dimension, by age and sex

The more prevalent chronic diseases were: pulmonary problems, hypertension, and back pain. E.Q. dimension with more problems (levels 2 and 3 of E.Q. 5D-3L- Fig. 1) was pain/discomfort (48.6%). One-third of respondents related moderate or severe anxiety/depression. All dimensions had more problems for elderlies. Women had more problems in dimensions pain/discomfort and depression/anxiety.

Analysing the risk of having moderate/severe problems (Table 2) by each dimension (E.Q. levels 2 and 3), there is a higher and statistical significant risk” among people with 50 years old or more for all dimensions, varying from 2.5 (pain/discomfort in 50–54 years range) to 11.5 (mobility in 60–64 years range). There is less risk of problems for male sex in all dimensions and a critical difference in risk associated with educational level. Comparing ISCD 7–8 stratum to ISCD 0–2, we have found 85% fewer odds for self-care problems and 78% fewer odds for mobility problems. The dimension anxiety/depression had a non-statistical difference with small differences between educational ranges.

**Table 2:** Logistic regression model (outcomes: any problems in each dimension, covariates: demographic variables)

	Mobility OR (95% CI)	Self-care OR (95% CI)	Usual activities OR (95% CI)	Pain/discomfort OR (95% CI)	Anxiety/depression OR (95% CI)
Age					
18–19	Reference				
20–24	1.11 (0.55–2.24)	0.43 (0.09–1.92)	0.72 (0.42–1.24)	0.96 (0.73–1.27)	1.35 (0.97–1.88)
25–29	1.72 (0.89–3.33)	2.30 (0.76–6.91)	1.11 (0.67–1.82)	1.04 (0.79–1.37)	1.83 (1.33–2.51)
30–34	2.28 (1.20–4.34)	2.03 (0.67–6.20)	1.23 (0.75–2.02)	1.37 (1.04–1.80)	1.70 (1.24–2.35)
35–39	3.40 (1.81–6.38)	2.54 (0.85–7.56)	1.20 (0.73–1.99)	1.50 (1.13–1.98)	1.88 (1.36–2.59)
40–44	4.02 (2.15–7.51)	3.75 (1.29–10.88)	2.22 (1.38–3.57)	1.52 (1.15–2.01)	2.01 (1.45–2.78)
45–49	6.14 (3.32–11.37)	3.93 (1.36–11.38)	2.85 (1.78–4.54)	1.74 (1.31–2.31)	2.14 (1.55–2.96)
50–54	10.33 (5.62–18.98)	6.33 (2.23–17.95)	4.05 (2.55–6.44)	2.51 (1.88–3.34)	2.58 (1.86–3.56)
55–59	9.93 (5.38–18.36)	8.42 (2.98–23.79)	3.22 (2.00–5.18)	2.29 (1.70–3.08)	2.62 (1.88–3.65)
60–64	11.48 (6.16–21.39)	7.41 (2.58–21.31)	3.51 (2.15–5.71)	2.29 (1.67–3.14)	2.37 (1.67–3.37)
Sex					
Female	Reference				
Male	0.69 (0.59–0.82)	0.62 (0.47–0.83)	0.71 (0.60–0.84)	0.45 (0.40–0.50)	0.57 (0.51 0.64)
ISCED 2011*					
0–2	Reference				
3–4	0.70 (0.58–0.83)	0.44 (0.32–0.59)	0.69 (0.57–0.82)	0.93 (0.81–1.05)	0.88 (0.77–1.00)
5–6	0.35 (0.27–0.45)	0.15 (0.09–0.26)	0.43 (0.34–0.54)	0.56 (0.48–0.65)	0.86 (0.73–1.00)
7–8	0.22 (0.14–0.34)	0.15 (0.06–0.38)	0.32 (0.21–0.48)	0.37 (0.29–0.47)	0.84 (0.66–1.07)
City					
Rio de Janeiro	Reference				
Recife	1.13 (0.91–1.40)	1.51 (1.06–2.16)	1.27 (1.03–1.57)	1.15 (1.00–1.34)	1.10 (0.94–1.28)
Porto Alegre	1.03 (0.83–1.30)	1.47 (1.02–2.11)	1.17 (0.94–1.46)	0.98 (0.84–1.14)	1.26 (1.08–1.47)

*International Standard Classification of Education 0–2 from no formal to lower secondary education. 3–4 upper to post-secondary, 5–6 tertiary education, 7–8 post-graduation

The mean utility for the sample was 0.824 (Table 3), with an absolute reduction of 11,2% from the first stratum (18–19 yrs) to the last (60–64 yrs). Additional data with utilities calculated EQ-5D index (time trade-off value set) and Visual Analytic Scale (VAS) stratified by sex and age is available [see Additional file 1] and [Additional file 2] respectively. The complete dataset is available [see Additional file 3].

**Table 3:** Mean utility values stratified by age group

Age	N	Mean	Sd	Min	p25	p50	p75	Max
18–19	321	0.873	0.13	0.508	0.787	0.801	1	1
20–24	675	0.876	0.13	0.302	0.787	0.801	1	1
25–29	747	0.859	0.148	0.061	0.787	0.801	1	1
30–34	728	0.85	0.151	0.114	0.737	0.801	1	1
35–39	649	0.834	0.155	0.305	0.737	0.787	1	1
40–44	615	0.817	0.179	− 0.176	0.737	0.787	1	1
45–49	613	0.801	0.182	− 0.131	0.731	0.787	1	1
50–54	574	0.765	0.187	0.068	0.64	0.787	1	1
55–59	487	0.762	0.198	− 0.028	0.634	0.787	1	1
60–64	365	0.761	0.212	− 0.176	0.634	0.787	1	1
Total	5774	0.824	0.172	− 0.176	0.737	0.787	1	1

## Discussion

This paper summarizes the Brazilian population norms for the EQ-5D-3L. It was conducted with a substantial number of face-to-face interviews representing the preferences of the general Brazilian population. It provides a normative value fit for use in health-related quality of life research and economic evaluation of health care interventions. Some data, also include in this study, including only one Brazilian federative state, was published by Viegas Andrade and cols [26]. In the Latin American region, norms were published for Argentina [27], Uruguay [28], and Colombia [29].

Comparing the results by age groups with other countries [30], it is possible to highlight some patterns. The most affected dimension was pain/discomfort, and the less affected was self-care, similar to international data. The prevalence of reported anxiety/depression problems above 30% was higher than 17 of 20 countries. Only Hungary (35.2%), Slovenia (36.4%), and Thailand (47.4%) had a higher prevalence.

The mean utility value set cannot be directly compared with other countries due to the application of different algorithms for utility estimation, but the range of values was narrow, varying from 0.873 (18–19 years) to 0.761 (70–74 years).

The results showing the education level as a determinant of the prevalence of health problems and quality of life are not a novelty. Recent data [31] concerning health-related quality of life showed an association of education years, employment rate, and family support with a better quality of life. Education years have a clear association with other socio-demographic factors like income, access to health care, medicines and disease preventive measures, and even better nutritional habits.

These normative values can be used as a baseline for group comparisons on cost-effectiveness models. These values would reflect the mean expected utility value of the population. Beyond economic models, the Brazilian population norms can be used as a measure of the health and disease burden of the general population.

The present study represents a large sample size, representative of four important urban cities. The data collection method, via face-to-face interviews, was essential for Brazilian reality, a country with a high proportion of functionally illiterate, especially elderlies.

We faced some limitations compromising the external validity of the data. The sample does not include respondents older than 65 years due to fieldwork challenges. Generalizability to rural samples may be limited, and the E.Q. 5D 3L is not the most recent version of the questionnaire. There is no value set for EQ-5D-5L in Brazil.

## Conclusion

Utility values vary with age and gender, being higher for men and younger individuals. The external comparisons using the Euroqol questionnaire are quick, feasible, and informative, and therefore, an important instrument to measure health. The trends in utility values observed in our sample are comparable to what was observed in other studies.

## Supplementary Information

**Additional file 1**. Utilities stratified by sex.

**Additional file 2**. Utilities stratified by age.

**Additional file 3**. Complete database.

## Data Availability

The complete database is available in Additional file 1.
